# Primary malignant Brenner tumor of the ovary: a case report

**DOI:** 10.3389/fonc.2025.1746988

**Published:** 2026-01-14

**Authors:** Kun Hao, Feng Gao

**Affiliations:** Department of Pathology, Hebei Medical University Third Hospital, Shijiazhuang, China

**Keywords:** cancer, immunohistochemistry, malignant Brenner tumor, ovary, pathology

## Abstract

Ovarian Brenner tumor is a rare type of ovarian epithelial neoplasm, predominantly benign, with only a minority exhibiting borderline or malignant characteristics. Malignant Brenner tumors (MBTs) resemble invasive urothelial carcinoma and often coexist with benign or borderline Brenner tumors. Clinically, they frequently present with non-specific symptoms, including abdominal pain and weight loss. We report the case of a 53-year-old female who presented with a mixed echoic mass in the left adnexa identified by gynecological ultrasound. Histopathological and immunohistochemical analyses of the resected specimens confirmed the diagnosis of ovarian MBT. The patient was subsequently referred for adjuvant treatment. This case highlights the diagnostic challenge of MBT and underscores the critical role of immunohistochemistry (positive for GATA3 and p63, negative for Pax-8) in confirming the diagnosis and distinguishing MBT from its histological mimics.

## 1 Introduction

Ovarian Brenner tumor represents an uncommon type of ovarian epithelial neoplasm, predominantly benign, with malignant variants constituting approximately 1–5% of cases (1). These tumors typically arise from metaplasia of the ovarian surface epithelium and may occur at any age, although they are more prevalent in perimenopausal women aged 50 to 70 years (1, 2). Most patients are asymptomatic, and the tumors are often incidentally discovered following ovarian surgery performed for unrelated conditions. However, larger tumors may elicit symptoms such as abdominal distension or pain. Due to their rarity and subtle or atypical clinical presentations, Brenner tumors are commonly identified during routine physical examinations or surgical interventions for other pelvic disorders (1).

This report describes a rare case of ovarian MBT in a postmenopausal patient who presented with abnormal uterine bleeding without associated endometrial pathology. The imaging, histomorphological, and immunohistochemical features are discussed to enhance understanding of this entity.

## 2 Case presentation

**Figure 1:**
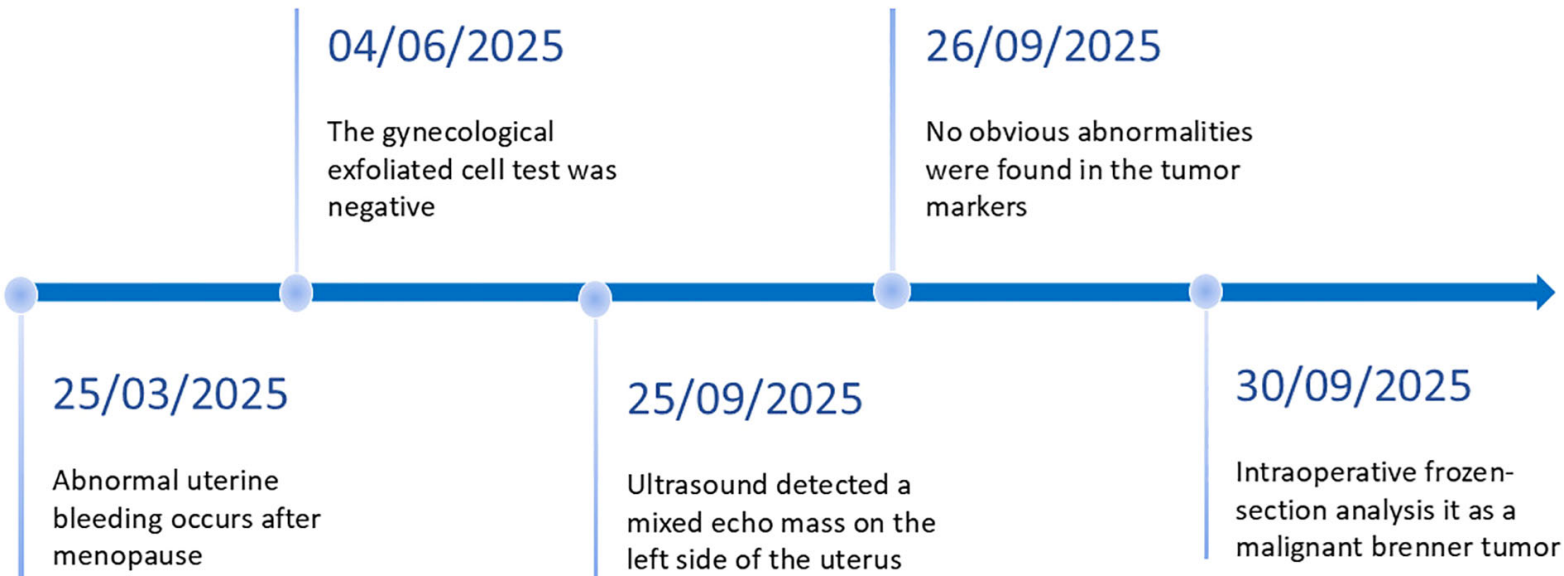
Representative timelines of patient’s relevant data history.

A 53-year-old woman presented with a six-month history of intermittent vaginal bleeding occurring two years after natural menopause. An initial transvaginal ultrasound performed at a local hospital revealed a 4.48 × 3.40 cm hypoechoic mass in the left adnexal region, exhibiting a heterogeneous internal echotexture and punctate hyperechoic foci. The patient subsequently consulted an external medicine department, where surgical intervention was recommended but declined. On September 28, 2025, she experienced recurrent minor vaginal bleeding and was evaluated at the gynecology outpatient clinic of the Third Hospital of Hebei Medical University (**Figure 1**).

**Figure 2:**
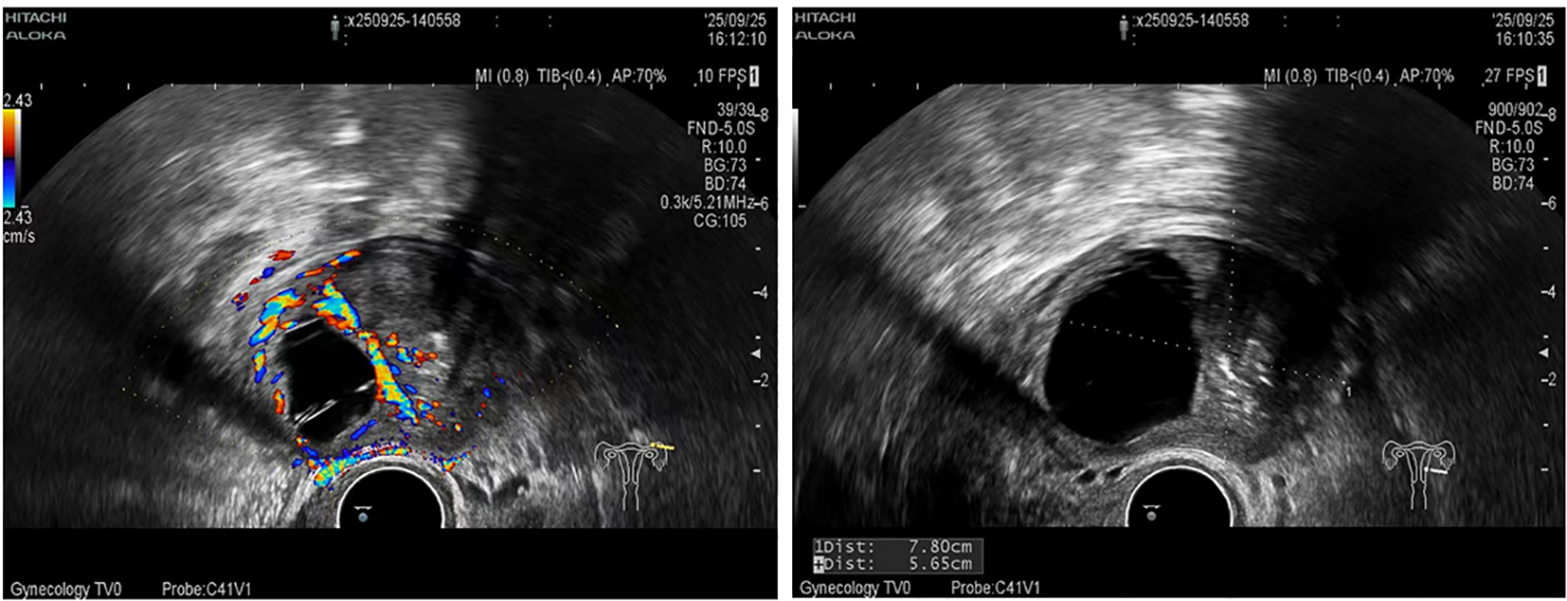
Ultrasound imaging demonstrating a mixed echogenic mass in the left adnexal region.

A follow-up transvaginal ultrasound demonstrated a 7.8 × 5.6 cm mass of mixed echogenicity adjacent to the left uterus. The cystic component contained multiple linear hyperechoic structures, while the local echoes in the solid area appeared uneven, with scattered punctate hyperechoic foci. The capsule was incomplete, with relatively clear boundaries. Doppler ultrasound indicated point-like linear blood flow signals within the solid region (**Figure 2**).

Upon admission, the physical examination revealed mild uterine enlargement. A non-tender, moderately mobile mass greater than 7 cm in diameter was palpated in the left adnexal region; the right adnexa was unremarkable. Laboratory investigations, including tumor markers (AFP, CEA, CA19-9, CA-125, CA15-3), reproductive hormones (LH, FSH, PRL, progesterone, estradiol, testosterone), complete blood count, and hepatic and renal function profiles, revealed no significant abnormalities. The patient was admitted for further diagnostic evaluation and management.

### 2.1 Therapeutic intervention

After admission, the patient underwent an endometrial biopsy. The biopsy result was negative. A laparoscopic procedure was performed on September 30, 2025, to evaluate the left adnexal mass of uncertain origin.

During the surgical procedure, a mass approximately 7 cm in diameter was observed encasing the left ovary. Subsequently, the mass, along with the entire uterus and bilateral fallopian tubes, was removed. Intraoperative frozen-section histopathology revealed a malignant mass in the left adnexa, suggesting a possible diagnosis of MBT. After communicating the findings to the patient’s family, comprehensive staging surgery for ovarian cancer was performed. This included right salpingo-oophorectomy, pelvic and para-aortic lymph node dissection, omentectomy, and multiple biopsies from pelvic, abdominal, and peritoneal sites. The patient recovered uneventfully post-surgery, experienced no significant complications, and was discharged in stable condition.

### 2.2 Histopathological and immunohistochemical findings

Macroscopic examination revealed a mass measuring 7.0 cm × 5.5 cm × 5.0 cm, characterized by cystic and solid components. The solid portion measured 5.0 cm × 4.0 cm and exhibited a grayish-white, fibrous appearance. In contrast, the cystic area featured a relatively smooth wall with a localized protrusion, and its thickness ranged from 0.2 to 0.3 cm.

Histologically, MBT resembled urothelial carcinoma, consisting of irregularly shaped and cytologically atypical transitional epithelial cells. Tumor margins were indistinct, and interstitial infiltration was noted. Tumor cells exhibited marked cellular atypia, variable nucleolar prominence, and abundant eosinophilic cytoplasm. The cystic regions were lined by multilayered epithelium, presenting features reminiscent of invasive urothelial carcinoma. Additionally, both benign and borderline Brenner cell nests were observed within the tumor tissue, along with intravascular tumor thrombi.

**Figure 3:**
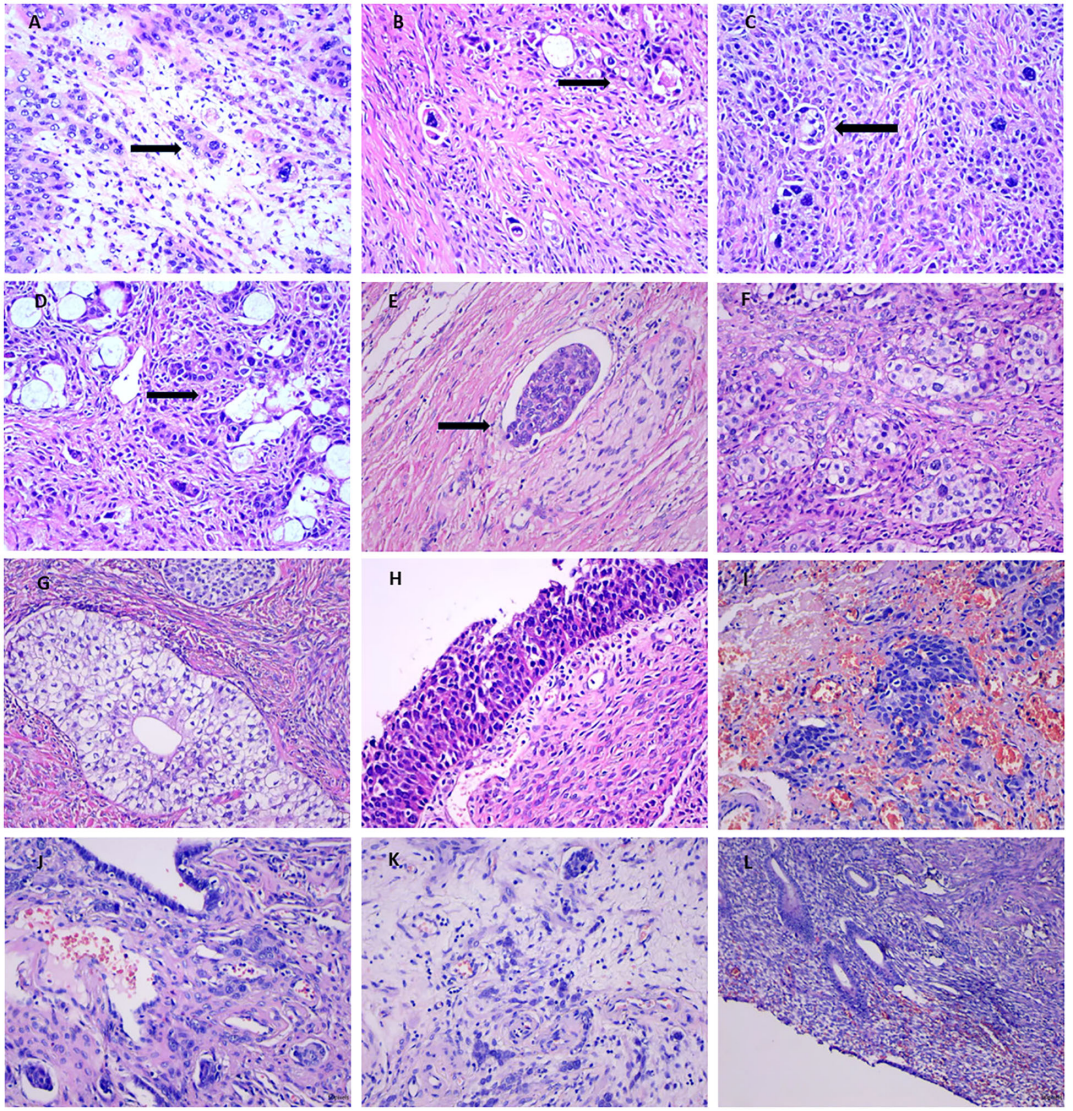
**(A)** Frozen section of MBT (×200; black arrow indicates malignant cells). **(B–D)** Hematoxylin and eosin (H&E) staining illustrating malignant cells in MBT (×200; black arrows indicate malignant cells). **(E)** H&E staining showing tumor thrombus (×200; black arrow indicates tumor thrombus). **(F)** H&E staining of borderline Brenner tumor areas (×200). **(G)** H&E staining of benign Brenner tumor areas (×200). **(H, I)** H&E staining of cystic tumor regions (×200). **(J)** Tumor metastasis in the left fallopian tube (H&E staining,×200). **(K)** Tumor metastasis in the right fallopian tube (H&E staining,×200). **(L)** H&E staining of endometrial tissue (×100).

Tumors were also identified on the uterine serosa and bilateral fallopian tubes, and atrophic endometrium was present. No evidence of tumor metastasis was detected in parametrial tissues, omentum, peritoneum, or lymph nodes (**Figure 3**).

**Figure 4:**
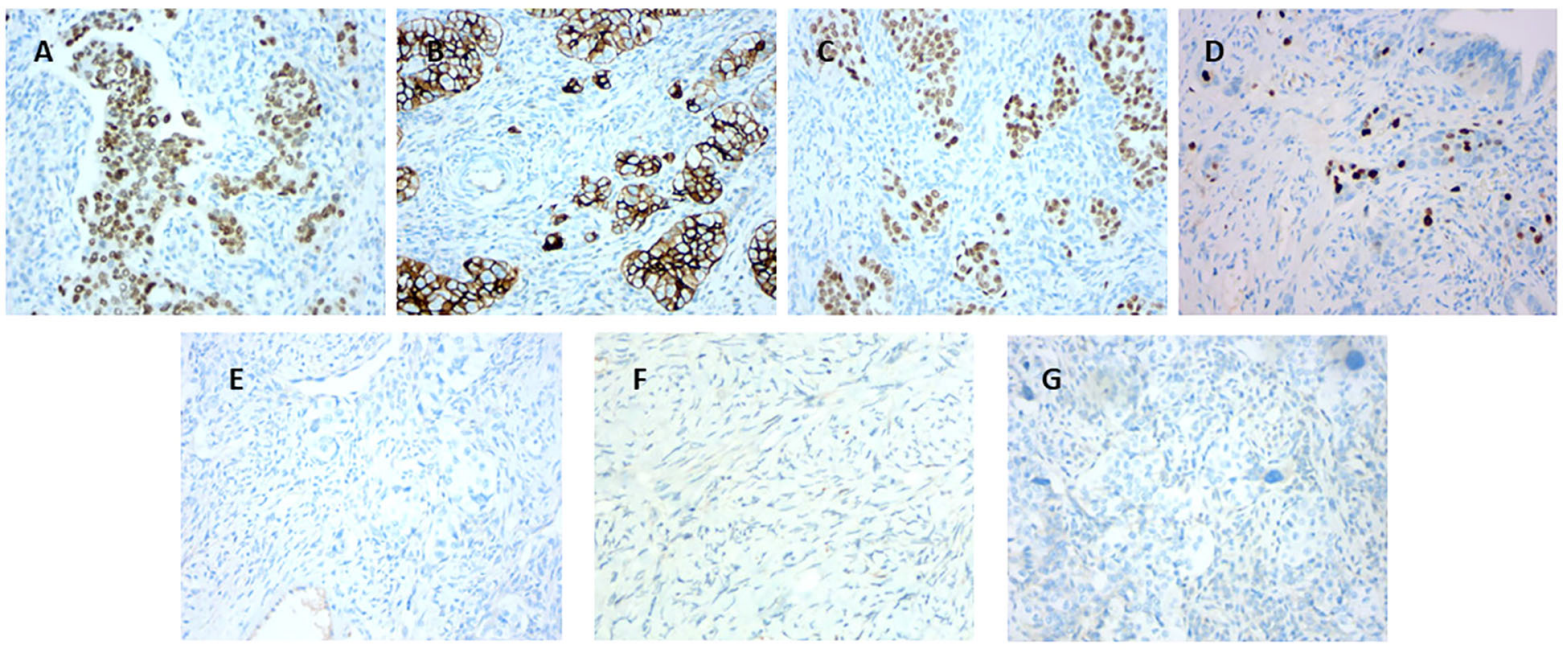
**(A)** Immunostaining demonstrating positive GATA3 expression (×200). **(B)** Immunostaining showing partial CK7 positivity (×200). **(C)** Immunostaining demonstrating positive p63 expression (×200). **(D)** Immunostaining for Ki-67 (hotspot area shows 40% positivity,×200). **(E)** Immunostaining for Pax-8 (negative,×200). **(F)** Immunostaining for inhibin-α (negative,×200). **(G)** Immunostaining for CK20 (negative,×200).

Immunohistochemical analysis demonstrated that Ki-67 was positive in hotspots (≥40%), CK7 (approximately 80%+), CR (approximately 40%+), EMA (approximately 20%+), GATA3(+), CAM5.2(+), and p63(+), whereas inhibin-α(-), p16(-), WT1(-), Pax-8(-), CK20(-), and Vimentin(-), and p53 was wild type (**Figure 4**).

### 2.3 Follow-up

The patient recovered well postoperatively without significant complications. The patient was diagnosed with FIGO stage IIA disease and is currently receiving adjuvant chemotherapy consisting of paclitaxel and carboplatin. Currently, there is no evidence of disease recurrence.

## 3 Discussion

The World Health Organization (WHO) classifies Brenner tumors into three categories: benign, borderline, and malignant. MBT of the ovary is a rare malignancy, representing a distinct subtype of ovarian cancer that resembles invasive urothelial carcinoma. It is commonly associated with benign or borderline Brenner tumors. Clinical manifestations commonly include abdominal mass or pain, and some patients report abnormal vaginal bleeding. Tumors are predominantly unilateral (>80%), although bilateral cases have been reported (1, 2).

In the present case, the patient’s chief complaint was postmenopausal abnormal uterine bleeding lasting six months. Previous studies indicate that approximately 4–14% of Brenner tumors are associated with endometrial hyperplasia, often linked to stromal luteinization and estrogen secretion, which may explain abnormal bleeding in some patients (3–5). Nevertheless, transvaginal ultrasound showed an endometrial thickness of 0.5 cm, and preoperative curettage revealed only blood clots with minimal endometrial tissue. Postoperative pathology confirmed an atrophic endometrium, and serum estrogen and progesterone levels were within normal ranges. These findings do not support estrogen-driven endometrial hyperplasia as the cause of bleeding. Further consideration of alternative mechanisms suggests a potential role for tumor-related factors. Vascular endothelial growth factor (VEGF) is known to promote angiogenesis, invasion, metastasis, and chemotherapy resistance in ovarian cancer (6), and elevated VEGF can increase vascular permeability. It is therefore conceivable that MBT may produce low levels of bioactive substances or growth factors that are not detectable by routine hormone assays. Such substances could be locally converted to active estrogen within endometrial tissue or induce bleeding by altering vascular permeability. Routine hormonal testing may not capture these tumor-derived mediators. Furthermore, atrophic endometrium itself is a well-recognized cause of postmenopausal bleeding (7). In postmenopausal women, marked estrogen deficiency leads to a thin endometrium with fragile vessels prone to rupture. Thus, abnormal bleeding may occur even when circulating hormone levels are within normal limits. This mechanism offers an additional plausible explanation for the bleeding observed in this patient.

Preoperative diagnosis of Brenner tumor remains challenging because its histological features closely resemble transitional epithelium. Morphologically, benign Brenner tumor cells show mild cytological atypia, with lightly stained cytoplasm, oval nuclei, and well-defined boundaries between cell nests and surrounding stroma. In contrast, MBT demonstrates indistinct nest boundaries with stromal invasion. Residual benign or borderline Brenner components are often present, which is consistent with the characteristic histopathological features of MBT.

Angel Yordanov et al. reviewed the literature and identified 10 reported cases of MBT, including ovarian and extra-ovarian types. Their results indicated that urothelial differentiation markers, such as GATA3, p63, and CK7, were predominantly positive, while Pax-8 was typically negative (8). The immunohistochemical results in our patient are consistent with their findings. Positive staining was observed for GATA3, CK7, and p63, with a Ki-67 labeling index exceeding 40% in hotspot areas. Postoperative pathological examination demonstrated metastatic lesions involving bilateral fallopian tubes and uterine serosa, further supporting the diagnosis of ovarian MBT. Histopathological and immunohistochemical analyses confirmed the diagnosis of MBT, and the differential diagnosis included the following entities:

### 3.1 Müllerian-derived tumors

Tumors arising from the Müllerian duct commonly present with abnormal uterine bleeding. Histologically, they are composed of large atypical cells with eosinophilic cytoplasm and frequently exhibit focal necrosis. PAX-8, a transcription factor typically observed in tumors of renal, thyroid, and Müllerian origin, is usually positive (9). The negative expression of PAX-8 in this case supports exclusion of Müllerian-derived tumors (8).

### 3.2 Urothelial-derived tumors

Histologically, malignant urothelial tumors can resemble MBT. CK20, an epithelial intermediate filament normally expressed in gastrointestinal and urothelial tissues, is detected in more than 90% of urothelial carcinomas (10). Negative CK20 staining and the coexistence of benign and borderline Brenner tumor components argue against urothelial origin in this case.

### 3.3 Metastatic gastrointestinal tumors

Ovarian metastases from gastrointestinal malignancies typically present at advanced disease stages and exhibit symptoms related to the primary gastrointestinal site. Microscopically, these tumors frequently display glandular or tubular formations with variable and irregular sizes. Tumor cells often exhibit diffuse infiltration and intracellular mucin production. CK20 is widely utilized as a marker for gastrointestinal tumors (11). In the present case, the absence of CK20 expression and lack of gastrointestinal symptoms exclude this diagnosis.

### 3.4 Sex cord-stromal tumors

These ovarian epithelial tumors consist of various subtypes and typically occur in younger patients. They often present with hormonal symptoms, such as hirsutism, virilization, menstrual irregularities, or precocious puberty (12). Inhibin-α, a member of the TGF-β family, is a key immunohistochemical marker used to identify sex cord-stromal tumors (13). The patient was postmenopausal without hormonal abnormalities, and negative inhibin-α staining supports exclusion of this tumor type.

For MBT, surgery remains the principal therapeutic approach. The surgical method selected depends primarily on tumor grade and clinical staging. Suitable procedures for appropriate patients include total hysterectomy, bilateral salpingo-oophorectomy, pelvic and/or para-aortic lymphadenectomy, random biopsies of peritoneal surfaces, and omentectomy (14). In the present case, comprehensive staging surgery for ovarian cancer was performed due to intraoperative frozen section results suggesting malignancy. Postoperative pathology showed no evidence of metastasis in lymph nodes or peritoneal tissues. However, owing to the rarity of MBT, chemotherapy regimens remain undefined, lacking well-established clinical guidelines. Previous studies have indicated potential therapeutic effects of paclitaxel combined with platinum-based chemotherapy, although recurrence rates appear relatively high (15). Nasioudis et al. analyzed clinical data from 202 MBT patients, demonstrating a five-year disease-specific survival of 94.5% in stage I patients, decreasing to 51.3% in patients at stage II or higher (16). In the present case, metastasis to the fallopian tubes and uterine serosa was identified, indicating a worse prognosis.

Thus, given the unique nature and rarity of this tumor, establishing comprehensive diagnostic and therapeutic protocols is crucial. These guidelines would facilitate early diagnosis, timely treatment, and individualized patient care, thereby improving patient outcomes and overall survival.

## 4 Conclusion

MBT is a rare tumor morphologically resembling urothelial carcinoma. This report described a patient with atypical symptoms preoperatively, and no significant abnormalities detected through tumor markers or conventional hormone assays. Although abnormal uterine bleeding is occasionally observed in Brenner tumor patients, the underlying causes remain uncertain. The present case proposes two possible mechanisms for such bleeding, both requiring further investigation.

In summary, this case highlights the necessity of further research into MBTs, particularly regarding pathogenesis, clinical features, and optimal therapeutic regimens. We emphasize the importance for clinicians to maintain a high index of suspicion for MBT, recommend regular health evaluations to patients, and ensure early diagnosis and management to enhance long-term patient survival.

## Data Availability

The original contributions presented in the study are included in the article/supplementary material. Further inquiries can be directed to the corresponding author.
